# Positive resources for combating depressive symptoms among Chinese male correctional officers: perceived organizational support and psychological capital

**DOI:** 10.1186/1471-244X-13-89

**Published:** 2013-03-19

**Authors:** Li Liu, Shu Hu, Lie Wang, Guoyuan Sui, Lei Ma

**Affiliations:** 1 Department of Social Medicine, School of Public Health, China Medical University, 92 North 2nd Road, Heping District, Shenyang, Liaoning, 110001, People's Republic of China; 2 Department of Basic Law, School of Forensic Medicine, China Medical University, 92 North 2nd Road, Heping District, Shenyang, Liaoning, 110001, People's Republic of China

**Keywords:** Depressive symptoms, Positive psychological capital, Perceived organizational support, Mediating role, Occupational psychology, Correctional officers

## Abstract

**Background:**

Although correctional officers (COs) clearly suffer from depression, positive resources for combating depression have been rarely studied in this population. The purpose of the study was to examine the associations of perceived organizational support (POS) and psychological capital (PsyCap) with depressive symptoms among Chinese COs.

**Methods:**

A cross-sectional survey was conducted in a province of northeast China during March–April 2011. A self-administered questionnaire was distributed to 1900 male COs from four male prisons. Depressive symptoms, POS, and PsyCap (self efficacy, hope, resilience, and optimism) were measured anonymously. A total of 1428 effective respondents with 953 frontline COs (FL-COs) and 475 non-frontline COs (NFL-COs) became our final sample. Hierarchical linear regression was performed to explore the factors associated with depressive symptoms. Asymptotic and resampling strategies were used to examine the mediating roles of PsyCap and its four components.

**Results:**

The level of depressive symptoms of FL-COs was significantly higher than that of NFL-COs (t = 2.28, p = 0.023). There were significant negative associations of POS, PsyCap, hope, resilience, and optimism with depressive symptoms among FL-COs. In NFL-COs, POS, PsyCap, and optimism were negatively associated with depressive symptoms. POS was positively associated with PsyCap and its four components among both FL-COs and NFL-COs. For FL-COs, PsyCap (a*b = −0.143, BCa 95% CI: –0.186, –0.103, p < 0.05), resilience (a*b = −0.052, BCa 95% CI: –0.090, –0.017, p < 0.05), and optimism (a*b = −0.053, BCa 95% CI: –0.090, –0.016, p < 0.05) significantly mediated the association between POS and depressive symptoms. For NFL-COs, PsyCap (a*b = −0.126, BCa 95% CI: –0.186, –0.065, p < 0.05) and optimism (a*b = −0.066, BCa 95% CI: –0.116, –0.008, p < 0.05) significantly mediated the association.

**Conclusions:**

Perceived organizational support and psychological capital could be positive resources for combating depressive symptoms in Chinese male COs. Psychological capital and its components (resilience and optimism) partially mediate the association between perceived organizational support and depressive symptoms. Therefore, organizational support and psychological capital investment (especially resilience and optimism) should be included in depression preventions and treatments targeting Chinese male COs.

## Background

Depression is common in the general population and is associated with extensive adverse effects in the workplace. In the European Union, 20% of the workforce suffers from some type of mental health disorder at any given time, particularly depression [1]. In China, with the rapid development of the society and economy, many workers face occupational mental health disorders. The average prevalence of depressive symptoms has been reported as 46.2% among various Chinese occupational populations including teachers, foreign enterprise employees, managers, researchers, traffic police, and community health workers [2]. The World Health Organization (WHO) estimates that depression will become the second-leading cause of disability worldwide by 2020 [3]. Depression has substantial adverse effects on workers’ behaviors, job performance, and quality of life [4-6].

Correctional officers (COs), working in a closed, coercive, and harsh prison environment, bearing multiple responsibilities for the correction, education and management of prisoners, and the maintenance of security and order inside prisons, are prone to suffer from various mental health disorders, especially depression [7-9]. In a French study, the overall proportion of COs with depressive symptoms was 24% [7]. Approximately one-third of the COs employed at two state prisons in the northeastern U.S. reported serious psychological distress [9]. This issue is particularly serious among Chinese COs because of high work demand and shortage of COs due to heavy prisoner load resulting from China’s huge population [10]. The ratio of COs to prisoners is 1:5 in China, which is lower than that in Western countries (1:3–1:4) [11]. As a result, COs are often required to work overtime, especially frontline COs (FL-COs) who have more contact with prisoners than other prison employees. Moreover, career advancement of COs is stunted by the relative disadvantage associated with lack of higher education [12]. Overall, COs’ efforts are not met with adequate reward [10]. Hu et al. [13] reported that the prevalence of depressive symptoms was 60.5% among Chinese COs. Depression can impact not only their career development and well-being, but also affect their functions related to correction and education that can help prisoners return successfully to society. Therefore, more research on the prevention and treatment of depression in Chinese COs is urgently needed. However, positive resources for combating depression have been rarely studied in this population.

According to organizational support theory [14], employees develop higher levels of perceived organizational support (POS) when their organization cares about their well-being and values their contributions. Consequently, employees with high POS are less likely to experience depression and anxiety [15,16]. As an important personal resource, the higher-order core construct of psychological capital (PsyCap) includes four state-like psychological capacities, including self efficacy, hope, resilience, and optimism, which can be measured, developed, and effectively managed [17]. PsyCap has significantly positive effects on employee performance, satisfaction, organizational commitment, and well-being in the workplace [17-19]. It is negatively related to job stress, cynicism, and burnout [20-22]. However, to our knowledge, the roles of POS and PsyCap in combating depression have not been studied among Chinese COs. Employees who perceive they work for more supportive organizations may be more likely to experience higher levels of PsyCap, which in turn has positive effects on their work behavior and job performance [23,24]. In addition, whether PsyCap mediates the association between POS and depression has not been confirmed. It is important to examine the positive effects of the four PsyCap components to develop specific preventions and treatments for depression because each component is conceptually and psychometrically distinct [25-27].

In light of the above concerns, the purpose of the present study was to verify the following three assumptions among Chinese COs: 1) POS as well as PsyCap and its components are negatively associated with depressive symptoms, 2) POS is positively associated with PsyCap and its components, and 3) PsyCap and its components mediate the association between POS and depressive symptoms.

## Methods

### Study design and sample

A cross-sectional survey was conducted in a province of northeast China during March–April 2011. The province comprises three metropolitan cities with more than 1,000,000 population, seven medium-sized cities with 500,000–1,000,000 population, and four small cities with 200,000–500,000 population. Based on geographic division and economic development, one metropolitan, two medium-sized cities, and one small city were selected. In China, the Ministry of Justice and the Bureau of Prison Administration supervise all correctional facilities and provide funds to ensure their normal operation (The Prison Law of the People’s Republic of China). After obtaining agreement from the provincial department of justice and prison superintendents, one male prison was selected from each city. The selected four prisons house prisoners with a sentence of more than 10 years. Female COs engage mainly in administrative and technical work in Chinese male prisons. Thus, male COs were our focus. We randomly sampled 50% of male COs from each department of selected prisons. In addition, these participants were divided into two occupational categories: FL-COs (working in direct contact with prisoners) and non-frontline COs (NFL-COs, including administrative staff and educational, technical, and healthcare workers). After the participants were given a brief and complete description of the study, written informed consent was obtained. A self-administered questionnaire was directly distributed to 1900 male COs. The sampled COs completed the questionnaire anonymously in a private place after their shift was over. Complete responses were obtained from 1428 individuals (response rate: 75.2%), of whom 953 (66.7%) were FL-COs and 475 (33.3%) were NFL-COs. The study was approved by the Committee on Human Experimentation of China Medical University, and the study procedures were in accordance with ethical standards.

### Measures

The Chinese version of the Center for Epidemiologic Studies Depression Scale (CES-D) was used as the indicator of depressive symptoms [28]. It consists of 20 items with 4 possible options that describe the frequency of respondents’ feelings in the past week ranging from 0 ‘rarely or none of the time (less than 1 day)’ to 3 ‘most or all of the time (5 to 7 days)’. The severity of depressive symptoms increases with a higher summed score and ranges from 0 to 60. The CES-D has been widely used among Chinese occupational populations with good reliability and validity [2,29]. In the present study, Cronbach’s alpha coefficient for the CES-D was 0.91 for FL-COs and 0.90 for NFL-COs.

POS was assessed using the 9-item version of the Survey of Perceived Organizational Support (SPOS) [14,30]. The scale consists of statements mainly concerning the organization’s valuation and well-being of employees. Each item is scored on a 7-point Likert scale ranging from 1 ‘strongly disagree’ to 7 ‘strongly agree’. In this study, the average score of the 9 items was used as the indicator of POS. Higher values indicated higher levels of POS. The 9-item SPOS has been extensively applied and validated among Chinese occupational groups [31,32]. Cronbach’s alpha for the POS scale was 0.89 for FL-COs and 0.88 for NFL-COs.

The Chinese version of the 24-item Psychological Capital Questionnaire (PCQ) was used to measure PsyCap [17]. The PCQ consists of four subscales: self efficacy (6 items), hope (6 items), resilience (6 items), and optimism (6 items). Each item has six responses with categories ranging from 1 ‘strongly disagree’ to 6 ‘strongly agree’. Average scores for the total scale and each subscale were calculated as indicators of PsyCap, self efficacy, hope, resilience, and optimism in this study, with higher scores indicating greater psychological capacities. Adequate reliability and construct validity of the PCQ has been demonstrated across multiple samples [17]. The Chinese PCQ has been initially and adequately applied among Chinese occupational populations [22,33,34]. For the total scale, alpha was 0.93 for both FL-COs and NFL-COs. Alpha for self efficacy, hope, resilience, and optimism subscales were 0.84, 0.86, 0.83, and 0.75 for FL-COs and 0.86, 0.86, 0.87, and 0.77 for NFL-COs, respectively.

The age, marital status, education, occupational category and monthly income (RMB) of COs were obtained in this study. Marital status was categorized as single/widowed/divorced or married/cohabiting. Education was categorized as junior college or lower and college or higher. Occupational category was categorized as FL-COs and NFL-COs. Monthly income was categorized as ≤ 3000 yuan, 3001–4000 yuan and > 4000 yuan.

### Statistical analysis

We analyzed the data for FL-COs and NFL-COs separately to allow for possible occupational differences in variable associations. Pearson’s chi-square (*χ*^2^) tests were used to compare differences in demographic characteristics between FL-COs and NFL-COs. Differences in continuous variables were examined by t-tests or one-way ANOVAs. Pearson’s correlation analyses were executed to examine the correlations among continuous variables. Univariate analyses were carried out to determine variables that were correlated with depressive symptoms with adjustment for age, marital status, education, and monthly income. The significant independent variables were then entered in the regression models. Hierarchical linear regression analysis was performed to explore the effects of groups of independent variables on depressive symptoms. In Block 1, age, marital status, education, and monthly income were added. POS was added in Block 2. There were two models (Model 1 and Model 2) in Block 3. PsyCap was added in Model 1, and self efficacy, hope, resilience and optimism were added in Model 2. Asymptotic and resampling strategies were used to examine the mediating roles (a*b product) of PsyCap and its four components on the association between POS and depressive symptoms, respectively [35]. In these equations, POS was modeled as the independent variable, CES-D score as the dependent variable, PsyCap and its components as the mediators, and age, marital status, education, and monthly income as covariates. The bootstrap estimate was based on 5000 bootstrap samples. Then, the bias-corrected and accelerated 95% confidence interval (BCa 95% CI) for each a*b product was investigated, and a BCa 95% CI not including 0 indicated a significant mediating role. All study variables were centralized before analysis to account for differences in scale scores. Moreover, tolerance and variance inflation factors were used to check for multicollinearity. All analyses were conducted using SPSS for Windows, Ver. 13.0. Statistical significance was defined as p < 0.05 (two-tailed).

## Results

### Characteristics of subjects

Demographic characteristics and differences in depressive symptoms are presented separately for FL-COs and NFL-COs in Table 1. NFL-COs were significantly more likely to be married (*χ*^2^ = 39.28, p < 0.001), had lower education (*χ*^2^ = 6.08, p = 0.014) and earned more (*χ*^2^ = 75.31, p < 0.001) than FL-COs. For FL-COs, married/cohabiting had a higher mean CES-D score than single/widowed/divorced (t = 2.29, p = 0.022), and those with junior college or lower education had higher CES-D score than those with college or higher education (t = 2.57, p = 0.010). For NFL-COs, those married/cohabiting had a lower mean CES-D score than those single/widowed/divorced (t = −2.59, p = 0.010).

**Table 1:** Demographic characteristics and differences in depressive symptoms

**Characteristics**	**FL-COs (N = 953)**			**NFL-COs (N = 475)**		
	**N**	**%**	**CES-D**	**N**	**%**	**CES-D**
			**Mean ± SD**			**Mean ± SD**
Marital status						
Single/widowed/divorced	250	26.2	17.71 ± 10.94	56	11.8**^a^	21.06 ± 12.85*
Married/cohabitation	703	73.8	19.59 ± 11.20*	419	88.2	17.25 ± 9.98
Education						
Junior college or lower	318	33.4*^a^	20.40 ± 10.60*	190	40.0	18.44 ± 10.22
College or higher	635	66.6	18.44 ± 11.38	285	60.0	17.20 ± 10.53
Monthly income (yuan)						
≤ 3000	355	37.3	18.00 ± 11.27	79	16.6**^a^	18.68 ± 12.40
3001–4000	522	54.8	19.69 ± 11.09	315	66.3	17.36 ± 10.24
> 4000	76	8.00	20.14 ± 10.83	81	17.1	18.06 ± 9.84

FL-COs: frontline correctional officers; NFL-COs: non-frontline correctional officers; CES-D: the Center for Epidemiologic Studies Depression Scale; POS: perceived organizational support; PsyCap: psychological capital.

^a^ Comparison between FL-COs and NFL-COs.

* p < 0.05, ** p < 0.01.

### Differences and correlations among continuous variables

Differences in continuous variables between FL-COs and NFL-COs and correlations among continuous variables are presented in Table 2. The average age of FL-COs (37.7 years) was significantly younger than that of NFL-COs (43.2 years) (t = 10.29, p < 0.001). The mean CES-D score of FL-COs was significantly higher than that of NFL-COs (t = 2.28, p = 0.023). The mean POS score of FL-COs was significantly lower than that of NFL-COs (t = −2.31, p = 0.021). However, there were no significant differences in PsyCap, self efficacy, hope, resilience, or optimism between FL-COs and NFL-COs. Age was positively correlated with CES-D score among FL-COs (r = 0.07, p = 0.037). CES-D score had negative correlations with POS, PsyCap, self efficacy, hope, resilience, and optimism among both FL-COs and NFL-COs.

**Table 2:** Differences and correlations among continuous variables

**Variables**	**FL-COs (N = 953)**		**NFL-COs (N = 475)**	
	**Mean ± SD**	**Pearson r with CES-D**	**Mean ± SD**	**Pearson r with CES-D**
CES-D	19.10 ± 11.16	–	17.70 ± 10.41*	–
Age	37.73 ± 9.80**	0.07*	43.22 ± 8.84	−0.01
POS	4.49 ± 1.23*	−0.41**	4.65 ± 1.15	−0.32**
PsyCap	4.31 ± 0.74	−0.42**	4.35 ± 0.72	−0.38**
Self efficacy	4.39 ± 0.85	−0.29**	4.40 ± 0.86	−0.27**
Hope	4.23 ± 0.90	−0.39**	4.27 ± 0.87	−0.34**
Resilience	4.36 ± 0.85	−0.36**	4.42 ± 0.84	−0.32**
Optimism	4.24 ± 0.90	−0.37**	4.28 ± 0.87	−0.36**

FL-COs: frontline correctional officers; NFL-COs: non-frontline correctional officers; CES-D: the Center for Epidemiologic Studies Depression Scale; POS: perceived organizational support; PsyCap: psychological capital.

* p < 0.05, ** p < 0.01.

### Associations of POS and PsyCap with depressive symptoms

All independent variables including POS, PsyCap, self efficacy, hope, resilience, and optimism were associated with depressive symptoms in univariate analyses with adjustment for age, marital status, education, and monthly income among both FL-COs and NFL-COs. Thus, they were entered in the hierarchical multiple regression models of FL-COs and NFL-COs.

Hierarchical multiple regression analysis results are presented in Table 3. For FL-COs, POS was significantly and negatively associated with depressive symptoms in Block 2. In Block 3 Model 1, POS and PsyCap were significantly and negatively associated with depressive symptoms. In Block 3 Model 2, POS, hope, resilience, and optimism were significantly and negatively associated with depressive symptoms. For NFL-COs, POS was significantly and negatively associated with depressive symptoms in Block 2. In Block 3 Model 1, POS and PsyCap were significantly and negatively associated with depressive symptoms. In Block 3 Model 2, POS and optimism were significantly and negatively associated with depressive symptoms. In addition, the effect of POS on depressive symptoms in Block 3 was reduced compared with that in Block 2 among both FL-COs and NFL-COs, as indicated by smaller β coefficients.

**Table 3:** Results from the hierarchical multiple regression analyses

	**Variables**	**Block 1 (β)**	**Block 2 (β)**	**Block 3 (β)**	
				**Model 1**	**Model 2**
FL-COs					
(N = 953)	Age	−0.015	−0.022	−0.034	−0.037
	Marital status	0.045	0.009	0.014	0.013
	Education	−0.070*	−0.045	−0.025	−0.028
	Monthly income 1	0.033	0.070	0.096*	0.099**
	Monthly income 2	0.046	0.054	0.078	0.080*
	POS		−0.405**	−0.263**	−0.257**
	PsyCap			−0.294**	
	Self efficacy				0.025
	Hope				−0.104*
	Resilience				−0.152**
	Optimism				−0.124**
	F	2.238*	32.993**	42.088**	31.043**
	Adjusted R^2^	0.006	0.168	0.232	0.240
	ΔR^2^	0.012*	0.161**	0.065**	0.075**
NFL-COs					
(N = 475)	Age	0.016	0.010	0.000	−0.002
	Marital status	−0.123*	−0.125*	−0.121*	−0.119*
	Education	−0.055	−0.051	−0.059	−0.056
	Monthly income 1	0.006	−0.011	−0.002	−0.001
	Monthly income 2	−0.005	−0.027	−0.025	−0.016
	POS		−0.327**	−0.201**	−0.204**
	PsyCap			−0.299**	
	Self efficacy				−0.093
	Hope				−0.040
	Resilience				−0.045
	Optimism				−0.195**
	F	1.726	11.102**	16.465**	12.152**
	Adjusted R^2^	0.008	0.113	0.186	0.190
	ΔR^2^	0.018	0.107**	0.073**	0.083**

FL-COs: frontline correctional officers; NFL-COs: non-frontline correctional officers; POS: perceived organizational support; PsyCap: psychological capital.

Marital status: married/cohabitation vs. single/widowed/divorced; Education: college or higher vs. junior college or lower; Monthly income 1: 3001–4000 yuan vs. ≤ 3000 yuan; Monthly income 2: > 4000 yuan vs. ≤ 3000 yuan.

There were two models (Model 1 and Model 2) in Block 3. PsyCap was added in Model 1, and self efficacy, hope, resilience and optimism were added in Model 2.

* p < 0.05, ** p < 0.01.

### Mediating effects of PsyCap and its components

Path coefficients a (between POS and mediators) and b (between mediators and depressive symptoms), a*b products, and BCa 95% CI for these products are presented in Table 4. For both FL-COs and NFL-COs, POS was significantly and positively associated with PsyCap, self efficacy, hope, resilience, and optimism. Consistent with the results from hierarchical multiple regression, PsyCap, hope, resilience, and optimism were significantly and negatively associated with depressive symptoms after controlling for age, marital status, education, monthly income, and POS among FL-COs. Thus, significant mediating roles of PsyCap (a*b = −0.143, BCa 95% CI: –0.186, –0.103), resilience (a*b = −0.052, BCa 95% CI: –0.090, –0.017), and optimism (a*b = −0.053, BCa 95% CI: –0.090, –0.016) on the association between POS and depressive symptoms were revealed among FL-COs. For NFL-COs, PsyCap and optimism were significantly and negatively associated with depressive symptoms. Thus, PsyCap (a*b = −0.126, BCa 95% CI: –0.186, –0.065) and optimism (a*b = −0.066, BCa 95% CI: –0.116, –0.008) significantly mediated the association between POS and depressive symptoms.

**Table 4:** Mediating roles of PsyCap and its components on the POS-depressive symptoms association

	**Mediators**	**a**	**b**	a*b (BCa 95% CI)
FL-COs				
(N = 953)	PsyCap	0.485**	−0.294**	−0.143* (−0.186, −0.103)
	Self efficacy	0.358**	0.025	0.009 (−0.031, 0.042)
	Hope	0.500**	−0.104*	−0.052 (−0.106, 0.001)
	Resilience	0.342**	−0.152**	−0.052* (−0.090, −0.017)
	Optimism	0.425**	−0.124**	−0.053* (−0.090, −0.016)
NFL-COs				
(N = 475)	PsyCap	0.423**	−0.299**	−0.126* (−0.186, −0.065)
	Self efficacy	0.230**	−0.093	−0.021 (−0.060, 0.012)
	Hope	0.491**	−0.040	−0.020 (−0.115, 0.066)
	Resilience	0.358**	−0.045	−0.016 (−0.068, 0.029)
	Optimism	0.339**	−0.195**	−0.066* (−0.116, −0.008)

PsyCap: psychological capital; POS: perceived organizational support; FL-COs: frontline correctional officers; NFL-COs: non-frontline correctional officers; BCa 95% CI: the bias-corrected and accelerated 95% confidence interval.

a: associations of POS with PsyCap and its components; b: associations of PsyCap and its components with depressive symptoms after controlling for the predictor variables; a*b: the product of a and b.

Age, marital status, education, and monthly income were covariates.

* p < 0.05, ** p < 0.01.

The proportion of total effect of POS on depressive symptoms by mediator role was calculated with the formula ‘(a*b)/total effect’. For FL-COs, the proportions of mediating roles of PsyCap, resilience, and optimism were 35.3%, 12.8%, and 13.1%, respectively. For NFL-COs, the proportions of mediating roles of PsyCap and optimism were 38.5% and 20.2%, respectively.

## Discussion

Findings show that in China, male COs seriously suffer from depressive symptoms, and POS and PsyCap can be positive resources for combating these symptoms. This is the first study to confirm the mediating roles of PsyCap and its components on the association between POS and depressive symptoms.

The prevalence of at least mild to moderate depression was quite high, as 59.7% of FL-COs and 56.2% of NFL-COs scored above 16 on the CES-D [2,29,36]. The level of depressive symptoms in our sample was higher than that of various occupational groups in Shanghai [2] and approximately 1.5 times higher than that found in the Chinese male general population [37]. The prevalence was also higher than that of male COs (24.9%) in France [7].

In addition, the level of depressive symptoms in FL-COs was higher compared with NFL-COs. Previous studies have shown that FL-COs suffer more serious mental health disorders than NFL-COs that can be attributed to occupational differences [38-40]. FL-COs and NFL-COs represent two significantly different work groups, despite working at the same place [39]. FL-COs work night shifts and spend almost all their working time in direct contact with the criminals. They bear great responsibility for prisoner correction as well as prison safety and control, and often face more stressors than NFL-COs in the workplace. NFL-COs have less direct contact with the criminals and fewer night shifts as well as more flexibility in performing tasks. We also found significant differences in demographic characteristics and POS between FL-COs and NFL-COs. Therefore, potential occupational differences should be taken into account to assess and effectively deal with depressive symptoms. We analyzed the data for FL-COs and NFL-COs separately.

It has been suggested that POS decreases workplace stressors and has the potential for combating job-related burnout, anxiety, and depression [41]. Our finding that POS was negatively associated with depressive symptoms among both FL-COs and NFL-COs supports this. POS is a highly effective resource that can predict a wide range of positive work attitudes and outcomes [41], and may help employees avoid depression and other mental health disorders. The significant difference in POS might be one of the important explanations for the difference in depressive symptoms between FL-COs and NFL-COs.

Furthermore, the positive effects of PsyCap on work attitudes and outcomes can help employees combat job stress, cynicism, burnout, and depression. Components of PsyCap have positive associations with desirable work attitudes and work performance [25-27,42]. Some of them also have positive effects on individual’s emotional functions [21,43-46]. For FL-COs, hope, resilience, and optimism had negative associations with depressive symptoms. Hope may provide individuals a positive resource for anxiety while protecting against perceptions of vulnerability, uncontrollability and unpredictability [21,43]. Resilient individuals show more emotional stability when faced with adversity in a constantly changing workplace environment [44]. Optimism is associated with a positive outlook or attribution of success, which includes positive emotions and motivation [45]. Workers with higher levels of optimism are less likely to experience symptoms of stress in the workplace [46]. However, only optimism was negatively associated with depressive symptoms for NFL-COs. The different psychological environment related to occupational categories is one possible reason for this finding. In prison, FL-COs face more occupational stressors considered risk factors for depression than NFL-COs, such as long working hours, role conflict, personal safety, and effort-reward imbalance [39]. Moreover, mean age of FL-COs was significantly lower than that of NFL-COs in our current data. These younger employees may face more problems outside the workplace related to family and social life [2,47]. Compared with NFL-COs, FL-COs may need more comprehensive psychological capacities to accomplish their multiple work functions and effectively deal with negative psychological consequences.

This is the first study to confirm the mediating roles of PsyCap and its components on the POS-depressive symptoms association. The COs who perceive more POS at work may be more likely to experience higher levels of PsyCap, which in turn reduces their depressive symptoms. Among the PsyCap components, resilience and optimism partially mediated the association between POS and depressive symptoms in FL-COs, and only optimism partially mediated the POS-depressive symptoms association in NFL-COs. POS can create positive conditions for developing PsyCap [48]. Individuals who perceive a high level of organizational support feel confident and hopeful about their desired job goals and are more likely to engage in voluntary behaviors. In addition, organizational support can help employees deal with problems, and promote the development of optimism and self-attribution regarding personal accomplishment [48].

Findings from our study have practical implication for prevention and treatment of depressive symptoms among Chinese male COs because PsyCap can be developed in a variety of ways [49,50]. Continued investment in financial, human, and social capital may no longer be sufficient in the workplace. Investment in PsyCap may yield substantial positive returns beyond the traditional forms of capital investment [20,51].

As a composite of psychological capacities, the direct effect of PsyCap on depressive symptoms and its mediating role on the POS-depressive symptoms association were greater than the specific effects of PsyCap components among both FL-COs and NFL-COs. This result was consistent with a previous study that indicated the synergistic role of PsyCap components [52]. Therefore, investment in each component is necessary to achieve maximum positive outcomes.

Effective strategies should be implemented with Chinese male COs to improve POS and PsyCap, and to relieve depressive symptoms. Especially, resilience and optimism should be given more attention in PsyCap investment. Policy makers and managers should improve the level of POS in COs by establishing fair and impartial procedures, affirming the contribution of COs and giving adequate praise and awards, maintaining effective communication with COs, helping them with career planning, providing good working conditions, improving job autonomy, and caring about their well-being [41,53,54]. For self-efficacy improvement, managers should provide opportunities for COs to develop career interests and skills, experience success, and boost their confidence. To facilitate a sense of hope, managers should help COs set appropriate, specific, and challenging job goals, create multiple pathways to achieve them, and activate a reward system. To improve optimism, managers should encourage COs to regard past failures and setbacks as valuable experience, develop a positive attribution style, and enhance their ability to discover and pursue various opportunities. For resilience improvement, managers should encourage COs to fully use their existing personal resources, develop solutions to avoid or overcome obstacles, facilitate critical reflection, and get adequate rest when burnout occurs [48-50,55,56].

However, before conclusions can be drawn, limitations of this study must be acknowledged. First, our study used a cross-sectional design, making it impossible to draw causal relations among study variables. However, study hypotheses were built on a solid theoretical and research foundation. The results of our cross-sectional study need to be confirmed in prospective settings. Second, the study population comprised only Chinese male COs from prisons that house individuals with a sentence of more than 10 years, which may limit the generalization of the results to other Chinese prison populations. Finally, gender differences in the associations between variables remain unaddressed and need further examination.

## Conclusions

Chinese male COs suffer from severe depressive symptoms. POS and PsyCap could be positive resources for combating depressive symptoms, with POS also being a positive resource for developing PsyCap and its four components among both FL-COs and NFL-COs. In addition, PsyCap, resilience, and optimism partially mediate the association between POS and depressive symptoms in FL-COs. For NFL-COs, PsyCap and optimism partially mediate the POS-depressive symptoms association. Therefore, POS and PsyCap investment (especially resilience and optimism) should be included in depression preventions and treatments targeting Chinese male COs.

## Abbreviations

CES-D: The center for epidemiologic studies depression scale; FL-COs: Frontline correctional officers; NFL-COs: Non-frontline correctional officers; POS: Perceived organizational support; PsyCap: Psychological capital.

## Competing interests

The authors declare that they have no competing interests.

## Authors’ contributions

LL designed the research, carried out data analysis and wrote the paper. SH organized the investigation. LW provided guidance in study design and was the corresponding author of the paper. GYS and LM provided help in the data collection, data analysis, results interpreting and paper writing. All authors read and approved the final manuscript.

## Pre-publication history

The pre-publication history for this paper can be accessed here:

http://www.biomedcentral.com/1471-244X/13/89/prepub
